# The Aroma of Non-Fermented and Fermented Dry-Cured Meat Products: Savory and Toasted Odors

**DOI:** 10.3390/foods14050881

**Published:** 2025-03-05

**Authors:** Lei Li, Carmela Belloch, Mónica Flores

**Affiliations:** Institute of Agrochemistry and Food Technology (IATA-CSIC), Agustín Escardino Avenue 7, 46980 Paterna, Valencia, Spain; lilei@cqaa.cn (L.L.); belloch@iata.csic.es (C.B.)

**Keywords:** aroma, savory, toasted, dry-cured meat, fermented

## Abstract

Volatile chemicals containing nitrogen and sulfur as key odors in dry-cured meat products have extremely low odor thresholds. These compounds play an important part in the overall uniqueness and characteristic flavor of dry-cured meat products, contributing to savory and toasted aroma sensations, respectively. In this review, we define the different volatiles and aroma compounds related to the flavor of dry-cured meat products. Moreover, the main differences regarding volatiles, aromas, and flavor profiles from non-fermented and fermented dry-cured meat products are summarized. Comparisons using the same volatile extraction techniques revealed that dry loins contained the most sulfur- and nitrogen-containing compounds, while complex flavor and aroma compounds in fermented sausages were greatly impacted by the fermentation process. The screening and quantification of savory and toasted odors showed that methionol, dimethyl sulfide, and 2-methyl-3-(methylthio)furan were mainly reported in non-fermented products, whereas pyrazines were mainly detected in fermented meat products. Finally, the different mechanisms in the generation of savory and toasted aromas, including chemical reactions and biochemical reactions by microorganisms (bacteria, yeast, and molds), are discussed. These discussions will help to better understand the complex flavor of dry-cured meat products.

## 1. Introduction

Dry-cured meat products are widely consumed because they are tasty and easy to eat, mainly in Europe and Mediterranean countries as well as in Asian countries (China and Korea). Non-fermented dry-cured meat products including dry-cured ham and dry-cured loins are produced using whole muscle pieces. Fermented dry-cured meat products and fermented sausages go through a fermentation step mediated by microbial starters. Fermented sausages are produced by comminuting meat and fat and combining them with sugar, salt, spices, and curing chemicals to form a meat batter which is further inserted into casings [1]. The manufacturing of both types of products includes a dry-curing process. However, the aroma and specific odor notes of these products differ, especially regarding key odor notes of savory and toasted aromas, among many other odor notes that are present in the products. The presence of chemicals that produce savory (meaty) and toasted odors in meat products may result in an olfactory balance that produces the distinctive “cured” odor, as no other volatile ingredient is responsible for the special smell of “cured” meat. Considering this factor, there are not many studies regarding these odor notes; this review focuses on the key odor notes (savory and toasted) present in dry-cured meat products, based on their worldwide production and consumption. These compounds are mainly present in non-fermented and fermented products. Dry-cured ham and dry-cured loin are the most typical products of the non-fermented type, while dry-fermented sausages are categorized as fermented dry-cured meat products in the recent ISO 23854 (2021) “fermented meat products specification” [2]. Nevertheless, a new ISO standard for “Dry-cured ham specification” is under discussion, indicating the commercial impact of this product worldwide.

Non-fermented dry-cured meat products are frequently manufactured and consumed in Europe. For this reason, most scientific studies consider this region the main reference; with the increase in globalization and innovation, recent studies also show some development in Asian countries [3,4]. The physicochemical properties of these products, as well as their geographical area of origin and the pig breed used for manufacturing, are described in Table 1. The main difference between products is observed depending on the pig breed and drying time. The drying and ripening steps are represented together in the table as drying time, which fluctuates from 211 to 413 days for ham and 18 to 156 days for dry loins. The pH of non-fermented dry-cured meat products lies between 5.4 and 6.5 because they do not undergo a fermentation process led by lactic acid bacteria. In dry loins, microbial activity primarily occurs on the surface of the meat, and a long drying and ripening storage time results in a reduction in microbial activity, resulting in diminishing major aroma compounds. However, very few studies have reported this with the diversity of bacteria found in loins as shown in Table 1 [5,6].

It is essential to remark that several Asian authors have classified dry-cured ham as a fermented meat product [7,8,9]. However, European dry-cured hams are not considered fermented meat products. The differences among dry-cured ham consumption habits also show that Chinese dry-cured ham is used as a seasoning in Chinese cuisine [10] due to its salty, umami, and characteristic aroma. Therefore, it is used in cooking treatments to increase meaty and cooked ham notes [10]. On the other hand, European dry-cured ham has a characteristic flavor and it is a ready-to-eat product. It should be considered that the sensory attributes of dry-cured hams are impacted by many factors from animal production to processing [11]. In addition, the characteristics of sensory attributes depend on conditions during processing which are adapted to the type of raw material employed.

**Table 1:** Main characteristics of non-fermented dry-cured meat products from different geographical origins.

Product	Geographical Area	Pig Breed	Physico-Chemical Properties				Microbial Counts (log cfu/g)				
			Drying Time (d)	Moisture Content (%)	Water Activity (a_w_)	pH	TMB ^a^	LAB	CN-Stp	YM	EB
Ham [12]	Spain	White pig	211	22.8	nr	5.9	nr ^b^	3.3	5.1	4.7(Y), 1.7(M)	<1
Ham [13]	Spain	Domestic	360	61.6	nr	6.2	nr	nr	nr	nr	nr
Loin [14]	Spain	Large White females Landrace and Pietrain Males × Duroc	49	58.5	nr	nr	nr	nr	nr	nr	nr
Loin [15]	Spain	Iberian × Duroc	120	33.9–38.6	nr	nr	nr	nr	nr	nr	nr
Loin [16]	Spain	Torbiscal Iberian	100	nr	<0.9	nr	nr	nr	nr	nr	nr
Loin [17]	Spain	Landrace × Large	156	46.1–49.6	nr	nr	nr	nr	nr	nr	nr
Loin [18]	Spain	Landrace × Large	18	47	nr	nr	nr	nr	nr	nr	nr
Loin [19]	Spain	Torbiscal Iberian	100	nr	<0.9	nr	nr	nr	nr	nr	nr
Loin [20]	Spain	Commercial	40	47	0.932	nr	2.8–3.3	2.2–2.8	nr	nr	nr
Loin [21]	Spain	Chato Murciano	60	41.9, 51.8	nr	nr	nr	nr	nr	nr	nr
Loin [22]	Spain	Celta breed (Barcina line)	90	34.04	0.841	5.8	nr	nr	nr	nr	nr
Loin [23]	Spain	Chato Murciano	60	46.01	nr	nr	nr	nr	nr	nr	nr
Loin [24]	Spain	Iberian	77	nr	0.875–0.883	5.5–5.7	nr	nr	nr	nr	nr
Loin [25]	Spain	50% Iberian × Duroc pigs	87	42.29	nr	nr	nr	nr	nr	nr	nr
Loin [26]	Spain	Iberian	80	45.7	0.92	5.6	5.2	nr	nr	4.4	nr
Loin [5]	Spain	Commercial	51	51.2–57.3	nr	5.5–5.6	5.3	4.1	5.4	5.0	nr
Loin [27]	Spain	Berkshire; Landrace × Yorkshire × Duroc	30	59.6–53.2	nr	6.2–5.9	nr	nr	nr	nr	nr
Loin [28]	Poland	Polish Large White	28	53.0	0.929	6.0	nr	nr	nr	nr	nr
Loin [29]	Poland	Puławska × Polish Landrace	118	nr	0.938–0.941	5.8–6.2	7.1–8.3	6.7–7.9	nr	nr	nr
Loin [30]	Poland	Polish Large White × Polish Landrace	nr	52.5	nr	5.9	nr	nr	nr	nr	nr
Loin [31]	China	Commercial Chinese	18	47.9	0.849	nr	nr	nr	nr	nr	nr
Jinhua Ham [32]	China	Large White × Landrace	263	31.9	nr	6.1	nr	nr	nr	nr	nr
Ham [33]	Korea	Korean native	413	39.1	0.84	6.5	nr	nr	nr	nr	nr

^a^ Total mesophilic bacteria (TMB), lactic acid bacteria (LAB), Gram-positive coagulase-negative staphylococci (CN-Stp), Enterobacteriaceae (EB), and yeasts and molds (YM). ^b^ nr: not reported.

The differences regarding the processing methods for producing fermented dry-cured meat products can vary greatly regarding raw material origins, spices, starter strains, casing, temperature, surface molds, smoking, etc. A compilation of the main features describing dry-cured fermented sausages from many parts of the globe is listed in Table 2. The most distinct sausage type originating in Europe is Sucuk, which is a type of Turkish sausage [34] made of beef. In contrast, most European fermented sausages are manufactured using lean and back fat from pork. Fermented sausages in southern Europe (Spain, Portugal, and Italy) are characterized by longer drying times in their manufacture, while shorter times, often less than a month, are used to produce sausages in northern Europe (Belgium and Norway) and Asian countries (China). Further differences between sausage manufacturers can be found concerning the duration of the drying time. Regarding physicochemical parameters, the pH of sausages is typically above 5.0, especially in southern European countries and China, although exceptionally lower values can be found in fermented sausages produced in northern European countries (Belgium). The pH of fermented sausages is related to the amount and type of added carbohydrates, the starter culture, and the fermentation conditions [35]. An additional significant feature of dry-fermented sausages, which can be kept for a long time due to their low a_w_ values (0.90), is shelf-stability. Fermented sausages are usually produced with the aid of a bacterial starter usually composed of lactic acid bacteria and staphylococci (Table 2). The bacterial counts in fermented sausages range from 6 to 8 log cfu/g of LAB and 4 to 6 log cfu/g of CN-Stp. The 2-log range variation in the bacterial counts found in fermented meat products has been attributed to the use of different bacterial starters and differences in the production techniques [36]. Yeast and fungi naturally grow on the surface of fermented sausages; therefore, differences in the manufacturing processes have a large effect on yeast and fungi counts in fermented sausages [37]. Besides beneficial microbial strains, spoilage bacteria (Enterobacteriaceae) have been detected in most fermented sausages (Table 2). The detection of undesirable bacteria indicates a deteriorating process, which would negatively affect a sausage’s quality and safety.

Dry-cured meat products have outstanding flavor and palatability due to proteolysis, lipolysis, and the formation of flavor compounds. The main factors that give these products a unique flavor are the ripening conditions and the meat quality. The primary distinction between fermented and non-fermented dry-cured meat products is the fermentation of carbohydrates during the production of fermented dry-cured meat products because of the starter culture’s action on sugar [38]. The addition of carbohydrates among other ingredients (salt, curing salts, etc.) and the fermentation conditions are essential for selecting the microbiota that contributes to the flavor of fermented sausages [28]. The addition of spices is also crucial in the meat products’ aroma [1], as it contributes to differentiating the characteristics of aroma.

**Table 2:** Main characteristics of fermented dry-cured meat products from different geographical origins.

Products	Geographical Area	Physico-Chemical Properties				Microbial Counts (log cfu/g)						
		Drying Time (d)	Moisture Content (%)	Water Activity (a_w_)	pH	TMB ^a^	LAB	CN-Stp	CP-Stp	EC	EB	YM
Fuet [39]	Spain	53	nr ^b^	0.619	5.60	8.59	8.30	4.62	nr	nr	nd ^c^	nr
Salchichón [40]	Spain	90	30.62	0.785	5.27	nr	7.61	4.52	nr	nr	nr	nr
Salchichón [41]	Spain	48	31.38	0.89	5.88	8.38	8.26	6.78	4.89	nr	3.12	nr
Chorizo [42]	Spain	48	20.66	0.821	5.58	8.59	8.17	nr	nr	nr	nr	6.23
Galician chorizo [43]	Spain	30	nr	0.830	6.13	8.55	8.51	6.38	nr	nr	5.61	nr
Dry-fermented sausages [44]	Spain	62	40.6	0.883	5.05	7.1	6.2	1.4	3.2	nr	nr	nr
Smoked fermented sausages [45]	Portugal	nr	nr	0.876	5.12	7.26	7.32	4.19	nr	nr	0.86	0.88 (Y), nd (M)
Fermented sausages [46]	Portugal	30–40	nr	0.85	5.32	nr	6.47	5.59	nr	2.73	nd	nr
Sicilian salami [47]	Italy	90	26.8	0.81	6.37	nr	7.11	6.11	nr	nr	0.15	5.16 (Y)
Dry-fermented sausages [48]	Italy	30	nr	0.794	5.77	4.5	8.0	5.6	nr	nr	5.0	3.4 (Y)
Vallo di Diano [49]	Italy	38	22.42	0.801	6.18	nr	8.53	7.61	nr	6.55	1.95	4.93
Salame nostrano [50]	Italy	21	23.96	0.807	5.66	8.01	7.00	6.16	nr	6.02	4.18	nr
Low-acid sausages [51]	Italy	100	nr	0.874	5.57	nr	8.30	4.97	nr	nr	nr	nr
Felino-type sausages [52]	Italy	42	nr	0.958	5.4	nr	7.50	nr	5.87	5.04	<1	3.13 (Y)
Dry-fermented sausages [53]	Italy	28	nr	0.875	4.92	nr	7.49	nr	5.62	4.06	nr	nr
Dry-fermented sausages [54]	Italy	60	nr	0.906	5.77	nr	8.55	nr	6.59	2.34	3.16	nr
Dry-fermented sausages [55]	France	60	nr	0.799	5.32	nr	6.69	5.32	nr	nr	nr	nr
Belgian-type salami [56]	Belgian	21	nr	0.925	4.81	8.71	8.71	5.91	nr	2.22	nd	1.58 (Y)
Boulogne sausages [56]	Belgian	28	nr	0.918	4.74	8.17	8.36	5.29	nr	1.70	nd	3.02 (Y)
Salami [57]	Norway	28	nr	0.829	5.28	nr	7.58	nr	nr	nr	nr	nr
Suçuk [34]	Turkey	16	nr	0.813	5.08	8.57	8.82	6.49	nr	nr	2.03	nr
Dry-fermented sausages [58]	China	18	nr	0.874	5.43	7.90	6.76	6.48	nr	nr	nr	nr
Harbin dry sausages [59]	China	12	25.54	0.784	5.33	nr	6.82	5.84	nr	nr	nr	nr
Dong fermented pork (Nanx Wudl) [60]	China	22	nr	0.877	5.22	nr	8.21	6.43	nr	nr	3.21	nr
Dry-fermented sausages [61]	China	23	nr	nr	5.24	7.97	7.97	6.85	nr	nr	5.25	4.82 (Y)
Dry-fermented sausages [62]	China	12	nr	nr	5.51	nr	7.53	6.72	nr	nr	5.52	nr

^a^ Total mesophilic bacteria (TMB), lactic acid bacteria (LAB), Gram-positive coagulase-negative staphylococci (CN-Stp), Gram-positive coagulase-positive staphylococci (CP-Stp), enterococci (EC), Enterobacteriaceae (EB), and yeasts and molds (YM). ^b^ nr: not reported. ^c^ nd: not detected.

## 2. Volatile Chemical Composition of Dry-Cured Meat Products

Aroma precursors found in the formulation of meat products determine the volatile composition of the final products [63]. Volatile organic compounds (VOCs) are mainly produced during the dry-curing step by chemical or enzymatic reactions like Strecker degradation, Maillard reactions, lipolysis, proteolysis, and chemical or enzymatic oxidation in dry-cured meat products [64].

**Figure 1:**
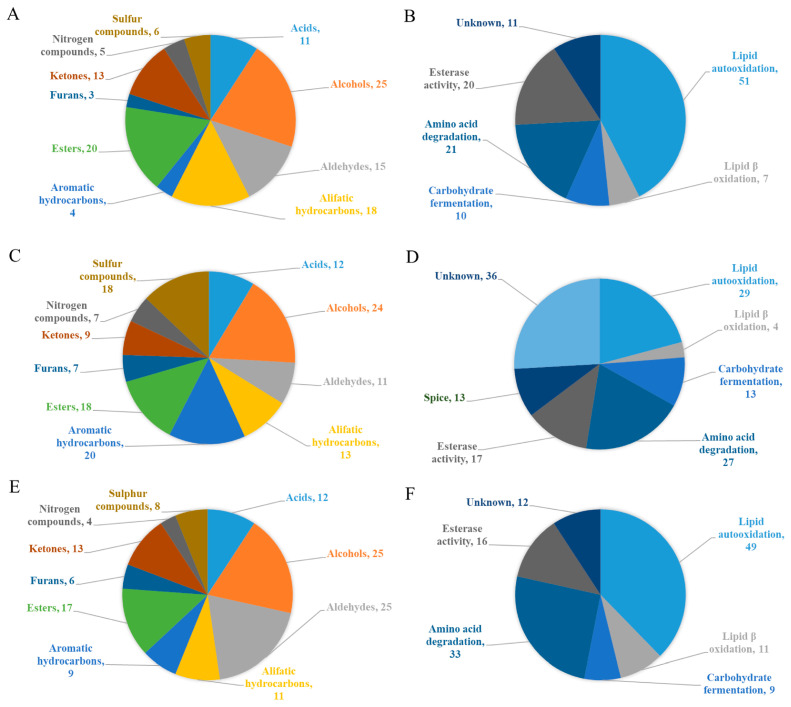
Frequency of volatiles detected by SPME (CAR/PDMS), where (**A**,**C**,**E**) are grouped according to probable origin and (**B**,**D**,**F**) according to chemical composition, respectively, of dry-cured ham [13,67], dry-cured loins [68,69], and dry-fermented sausages [70,71].

The identification of volatile compounds is necessary for the aroma profile characterization of dry-cured meat products [65]. However, the process used for VOC extraction has a significant impact on the characteristics and odor profile of dry-cured meat products. Studies using CAR/PDMS fiber and headspace–solid-phase micro-extraction/gas chromatography–mass spectrometry (HS-SPME/GC-MS) have enabled the comparison of the volatile profile of fermented and non-fermented dry-cured meat products (Figure 1). In plots where the compounds are classified by their chemical origin, alcohols make up a significant portion of the compounds in dry-cured ham and loins, whereas aldehyde VOCs are mostly found in fermented sausages. Dry loins (Figure 1C) contain 18 sulfur-containing and 7 nitrogen-containing compounds, whereas dry-cured ham (Figure 1A) and dry-fermented sausages (Figure 1E) have modest numbers of compounds. According to their most probable origin, several of the volatile chemicals identified in dry-cured ham products are due to lipid autooxidation, closely followed by amino acid degradation and esterase activity, as occurs in ham (Figure 1B). However, differences are found in Chinese Jinhua ham, which is characterized by odor-active compounds resulting from the breakdown of amino acids, lipids, and carbohydrates through the Maillard reaction [66]. In addition, spices contribute to the aroma of dry-cured loins as much as carbohydrate fermentation. However, no clear origin has been found for numerous aroma compounds in dry-cured loins (Figure 1D). Finally, the main VOCs found in dry-fermented sausages originate from lipid autooxidation and amino acid degradation (Figure 1F).

## 3. Comparative Aroma and Flavor Profiles: Fermented vs. Non-Fermented Products

**Figure 2:**
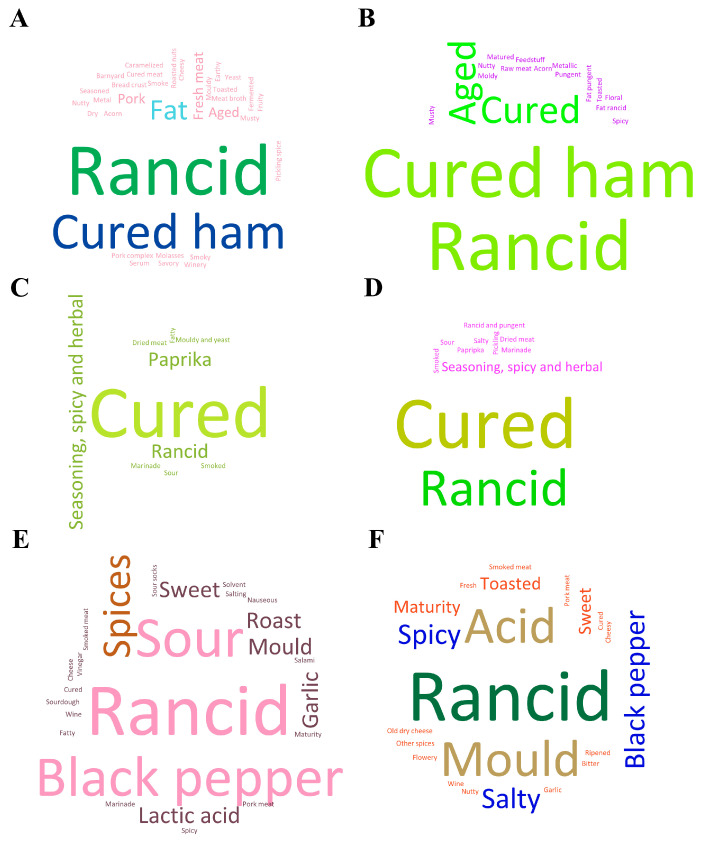
Word clouds of sensory descriptors used to describe the aroma (**A**,**C**,**E**) and flavor (**B**,**D**,**F**) of dry-cured ham (**A**,**B**) [75,76,77,78,79,80,81,82,83,84,85,86,87,88,89], dry-cured loins (**C**,**D**) [17,21,25,72,73,90,91,92,93,94], and dry-fermented sausages (**E**,**F**) [41,49,50,51,57,95,96,97,98,99,100], respectively.

Aroma is related to olfactory sensation, while flavor is an intricate blend of the trigeminal, gustatory, and olfactory senses experienced while tasting. The word clouds representing the sensory descriptors employed in various scientific publications (Figure 2) characterize the aroma (left) and flavor (right) of dry-cured meat products. The definitions used for several aroma (orthonasal) descriptors are listed in Table S1. Most descriptors used in Figure 2 were acquired using the sensory approach of Quantitative Descriptive Analysis (QDA), whereas others were acquired using the Flash Profile method [25] and Check-All-That-Apply (CATA) technique [72]. The plots show that rancid and cured ham are the main sensory descriptors in dry-cured ham (Figure 2A,B), while cured and rancid are especially indicative in dry-cured loins (Figure 2C,D) together with descriptors related to the addition of spices, such as spicy and herbal [73]. In contrast, fermented sausages, like salami from Italy and salchichón from Spain, have a very complex flavor and aroma (Figure 2E,F). The primary sensory descriptors used are rancid, sour, and black pepper, whilst rancid, acid, and mold define the aroma of fermented sausages. The application of starter cultures (LAB and CN-Stp) enhances the aroma profile and complexity of dry-cured fermented sausages. Specially, LAB inoculation allows the control of acidification, which slows down the synthesis of cured aroma compounds such as methyl ketones (2-pentanone, 2-hexanone, and 2-heptanone) [74].

## 4. Screening of Savory and Toasted Odors by Olfactometric Detection

Olfactometry and GC-sniffing have been used to identify the odor and sensory properties of volatile compounds in many natural products [101]. Previous studies have revealed that choosing a suitable sample preparation method (DHS: dynamic headspace; P&T: purge and trap; SPME: solid-phase micro-extraction; HSSE: headspace sorptive extraction) is very important for aroma screening [102]. Studies have also shown that not all volatile compounds are important for the aroma profile, but the determination of a volatile odor activity value (OAV) can provide insight into the beneficence of each compound to the overall odor. In the case of food, the ratio of the volatile compound’s content divided by its odor threshold in an appropriate matrix (water, oil, or air) is used as the OAV [101]. Regarding sample preparation techniques, the SPME method has been frequently employed in volatile compounds’ analysis. However, just a tiny percentage of the volatiles found in meat products made from dry-curing can be considered odor-active because their sensory properties depend on their concentration and interaction with the matrix composition [103].

**Figure 3:**
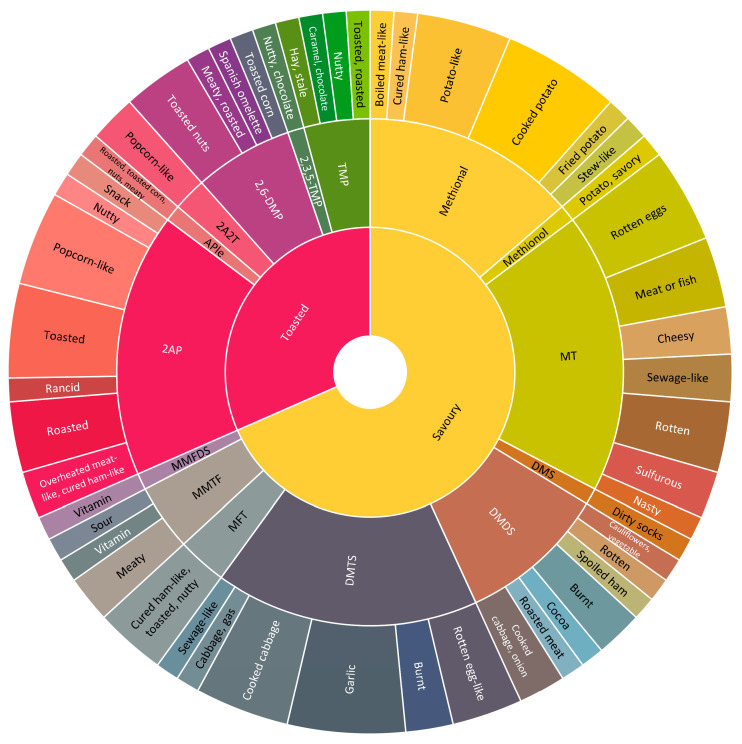
An odor wheel describing the aroma of non-fermented dry-cured meat products, including the main savory and toasted identified compounds. Data are based on the number of times a compound was reported [66,78,81,88,107,108,109,110,111,112,113,114,115,116]. Abbreviations of compounds: DMTS: dimethyl trisufide; APle: 2-acetylpyrrole; 2A2T: 2-acetyl-2-thiazoline; 2,6-DMP: 2,6-dimethylpyrazine; 2,3,5-TMP: 2,3,5-trimethylpyrazine; TMP: tetramethy-lpyrazine.

**Figure 4:**
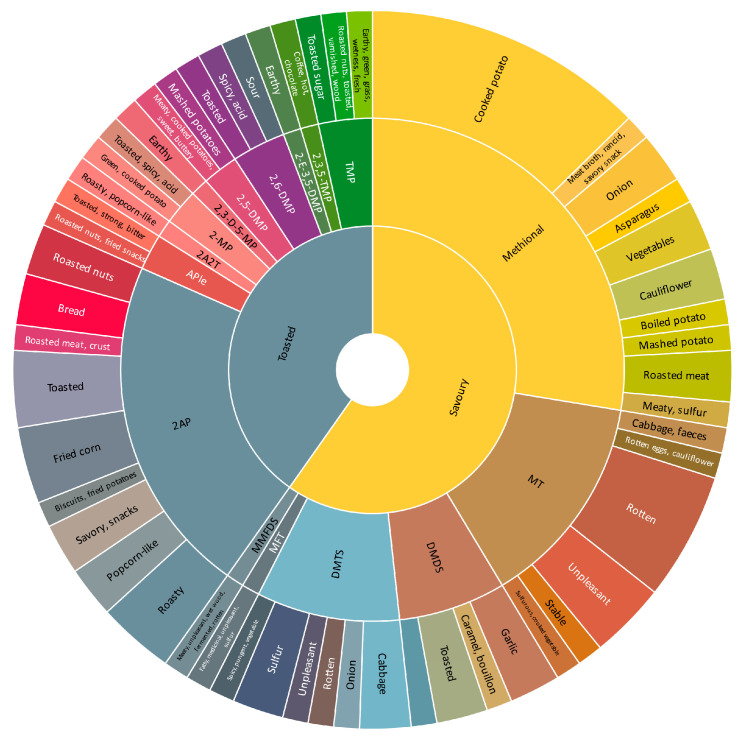
An odor wheel describing the aroma of fermented dry-cured meat products, including the main savory and toasted identified compounds. Data are based on the number of times a compound was reported [70,113,117,118,119,120,121,122,123,124,125,126,127,128,129,130,131,132]. Abbreviations of compounds: 2,5-DMP: 2,5-dimethylpyrazine.

The odor wheels in Figure 3 and Figure 4 are used to represent savory and toasted odor-active compounds, and their sensory descriptions were reported using Olfactometric Detection in non-fermented and fermented dry-cured meat products, respectively. The bibliographic references employed for these figures are indicated in the figure legends. The data are based on the number of times that the different compounds were reported in previous scientific studies. The compounds found are essentially similar in both wheels except for the presence of methionol, dimethyl sulfide (DMS), and 2-methyl-3-(methylthio)furan (MMTF) in non-fermented products, while several pyrazines (2-methylpyrazine (2-MP), 2,3-diethyl-5-methylpyrazine (2,3-D-5-MP), and 2-ethyl-3,5-dimethylpyrazine (2-E-3,5-DMP)) were found in fermented meat products. In most cases, a clear descriptor defined the aroma profile of the savory compounds in both fermented and non-fermented products. One of the compounds is methional, where the aroma was mainly (but not only) described as cooked potato. The same happened in the case of methanethiol (MT) and dimethyl disulfide (DMDS), where the aroma was associated with rotten notes and cooked cabbage, respectively, as shown in both wheels. Other important savory notes including meaty and nutty in non-fermented meat products were related to the presence of 2-methyl-3-furanthiol (MFT) and MMTF, whereas in fermented products, meaty notes were reported in the presence of methyl 2-methyl-3-furyl disulfide (MMFDS).

In the case of toasted aroma compounds, only 2-acetyl-1-pyrroline (2AP) was associated with toasted, roasted, and popcorn-like notes in both fermented and non-fermented products. Pyrazine compounds in non-fermented meat products mainly contributed to toasted notes, while in fermented products, different odor descriptors like earthy, mashed potatoes, etc., were reported. These differences in aroma notes described by Olfactometric Detection may be related to the content of various volatile compounds [104] and pyrazines, which have a synergistic impact on aroma perception [105]. Furthermore, recent studies have revealed the saltiness-enhancement properties of pyrazines and sulfur compounds present in cooked Jinhua dry-cured ham and their effect on flavor perception [106].

## 5. Mechanisms of Savory and Toasted Aroma Compound Formation

Clarifying the source and formation of toasted and savory odors in fermented dry-cured products is essential to understanding the impact of raw materials, ingredients, and processing. In cooked meat, meaty and sulfurous aromas are the primary source of savory odors [133]. These aromas originate from ribose and sulfur-containing amino acids through the Maillard reaction [134]. Other reactions, like thiamine degradation, also favor the formation of savory aromas in cooked meat, whereas toasted odors are mainly generated through a cooking process that produces toasted, roasted, and cooked meat odors. These odors are primarily formed by Maillard reactions involving reducing sugars and amino acids and also nitrogen compounds [135].

**Figure 5:**
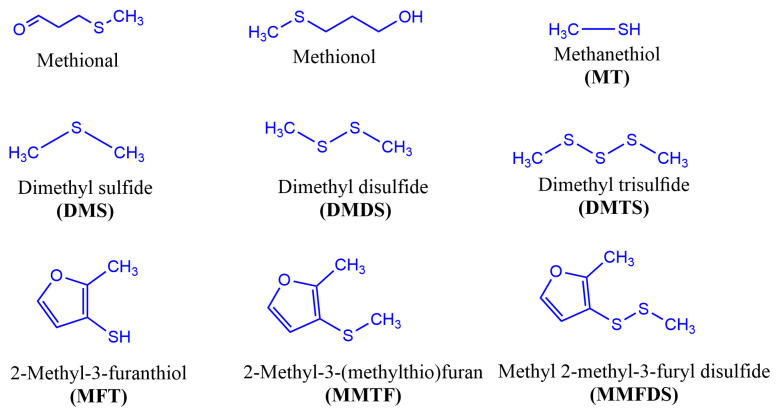
Chemical structure of savory aroma compounds in dry-cured meat products.

**Figure 6:**
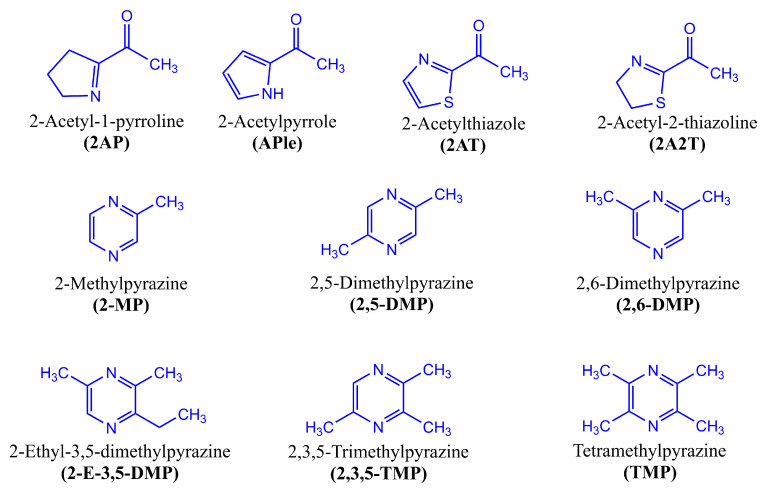
Chemical structure of toasted aroma compounds in dry-cured meat products.

In dry-cured meat products, the main chemical structures of compounds related to savory (Figure 5) and toasted aromas (Figure 6) are the result of different chemical and biochemical reactions. As previously reported, the savory aromas in fermented and non-fermented meat products are attributable to the presence of sulfur compounds (Figure 3 and Figure 4), which have low odor thresholds. In the case of toasted aromas, their presence is related to nitrogen heterocyclic compounds [66].

**Figure 7:**
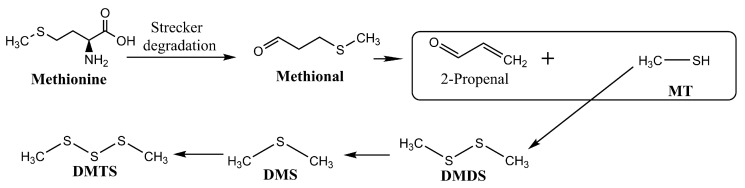
Chemical reactions that contribute the generation of savory aromas from methionine. Adapted from [135] with the publisher’s permission.

**Figure 8:**
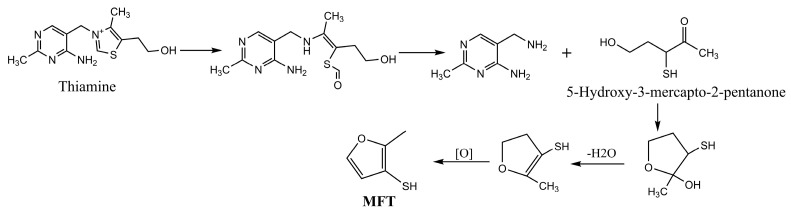
Chemical reactions involved in the generation of savory aromas from thiamine. Reprinted from [137] with the publisher’s permission (MDPI under the terms of the CC BY license).

The generation of savory aromas in dry-cured meat products is a result of several reactions. One of the main reactions may be the breakdown of methionine mediated by the Strecker reaction (Figure 7), which produces methional. Methional eventually breaks down into MT, which quickly oxidizes to DMDS. This compound is then transformed, becoming DMTS and DMS. Similarly, the degradation of thiamine can produce MFT (Figure 8). Although these reactions have been studied at high temperatures, as occur in cooked meat products, methionine degradation and methional generation have also been observed under mild-temperature conditions in a meat model simulating dry-fermented sausage [136], thus demonstrating that the generation of savory aromas can also occur in curing conditions.

**Figure 9:**
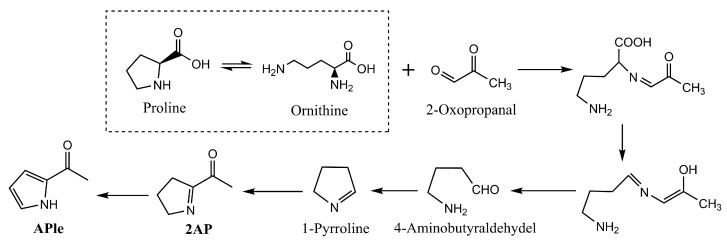
Chemical reactions involved in the generation of 2AP and APle from the reaction of proline and ornithine with 2-oxopropanal. Adapted from [142] with the publisher’s permission.

The generation of toasted aromas may be related to the presence of proline and ornithine, which have been identified as amino acids reacting with 2-oxopropanal, the Maillard reaction’s intermediate, to produce 2AP. The latter compound has been found to contribute to toasted aromas (Figure 9) in several processed foods, such as toasted wheat bread and cooked sweet corn [138]. However, 2AP is a very unstable compound, and at room temperature, it can be oxidized to form APle [139]. In dry-cured meat products, processing is performed at mild temperatures which do not allow the formation of 2AP from proline and ornithine through the Maillard reaction [140]. Nevertheless, the presence of yeasts and molds may mediate biochemical processes that could modulate the content of 2AP in dry-cured meat products [141].

In contrast with meat products submitted to thermal treatment [135], the mild environmental conditions applied in dry-cured processing are the limiting factor for savory and toasted odor compound formation. Therefore, the contribution of biochemical reactions mediated by autochthonous or inoculated microorganisms (bacteria, yeasts, and molds) to generate the savory and toasted aromas in fermented dry-cured meat products acquires special relevance. The importance of microbial activity and its impact on the generation of savory and toasted compounds has been previously demonstrated [1]. The species *Staphylococcus xylosus* [143] generates sulfur DMDS and methional from methionine and nitrogen compounds; moreover, 2,5-DMP from threonine in a chemically defined medium [144]. The potential of *Debaryomyces hansenii* in the savory and toasted aroma compound formation in dry-cured meat products has also been investigated intensively. In the production of dry-cured meat products, *D. hansenii* is the most prevalent and dominating yeast throughout the stages of fermentation and maturation [70,145,146,147]. However, not all *D. hansenii* strains isolated from fermented dry sausages have the same potential to produce volatile sulfur compounds (methionol, dimethyl sulfide, etc.) from sulfur-containing amino acids [148]. Previous studies have shown the importance of the selection criteria of *D. hansenii* strains with the ability to generate savory and toasted aromas [149]. The inoculation of *D. hansenii* in dry-cured hams and loins (Table S2) has been associated with the formation of savory compounds such as methional, MT, and DMDS. Meanwhile, in dry-fermented sausage, additional compounds of MFT, MMFDS, 2AP, 2A2T, and 2,6-DMP can also be generated. Regarding toasted odor compounds, 2,5-DMP, 2-E-3,5-DMP, and TMP have been found in dry-cured hams and loins, while 2AP, 2A2T, and 2,6-DMP have been found only in fermented sausages.

A study on Mediterranean sausages showed the generation of 2AP related to surface isolated *Penicillium nalgiovense* [150]. Moreover, 2AP has been found in Iberian ham [107], Jinhua ham [66,112], American country ham [115], and Chinese dry-fermented sausages [61]. Nevertheless, more studies are needed to demonstrate mold’s contribution to 2AP formation.

## 6. Discussion and Implications

Different concentrations of aroma-active chemicals give distinct flavors and aromas to fermented and non-fermented dry-cured meat [151]. The cured odor is most likely produced by a quantitative balance of savory and toasted odors because a single substance has not been found to be accountable for the distinct odor of dry-cured meat products [1]. The amount of previous research involving the accurate quantification of savory and toasted aromas is not very significant (Table 3). Moreover, the quantification of savory and toasted aroma compounds in meat products can be difficult, due to the low concentration of these substances and the complexity of the meat matrix [141]. In fermented products (sausages), several studies have reported the quantification of savory and toasted aroma compounds. However, only one study has reported compound quantification results in non-fermented products like dry-cured loins. Nevertheless, in all cases, the quantities reported for the aroma compounds were extremely low, in the range of µg/kg, except for methional and DMDS. Furthermore, in addition to the quantitation process, the procedures employed for volatiles’ extraction must be taken into account for comparisons between studies [152]. In fermented products, several studies have revealed the quantification of savory and toasted odors after extraction using the technique of solid-phase micro-extraction (SPME), except for two studies that used solvent-assisted flavor evaporation (SAFE) [119,132]. In the latter two studies, a number of compounds and low amounts of these compounds were reported. Moreover, high variability was observed in the compounds detected and quantities reported. However, these variations could be attributed to differences in meat product processing, the extraction techniques used to enrich the target compounds [120], and the reactivity of sulfur compounds [153]. Nevertheless, the quantification of savory and toasted flavor compounds is very important to studying their formation mechanism and their influence on the flavor of meat products; therefore, further research should concentrate on the quantification of these compounds in non-fermented dry-cured meat products.

The study of the reaction mechanism affecting the concentrations of savory and toasted volatile compounds in dry-cured meat products can improve organoleptic characteristics. In addition, this knowledge can be employed to control the drying process without losing savory and toasted aromas. Finally, long drying and ripening times can be optimized to increase the sustainability of the production process.

## 7. Conclusions and Future Directions

In summary, the presence of chemicals that produce savory (meaty) and toasted odors in meat products may create an olfactory equilibrium that leads to the distinctive “cured odor”, as no one compound is responsible for the unique odor of “dry-cured” meat. Depending on the chemical and biological events during the process, multiple strategies may be used to generate these savory and toasted flavor components. Consequently, investigating the mechanism that involves producing them in various dry-cured meat products—both fermented and non-fermented—helps to comprehend the influence of their aroma on these meat products. Knowledge of the mechanism of aroma formation can be used to develop savory (meaty) and toasted meat products and flavorings.

## Figures and Tables

**Table 3:** Content (µg/kg) of savory and toasted volatile compounds reported in dry-cured meat products.

Compounds	Loin [141]	Sausage [119]	Sausage [119]	Sausage [141] ^c^	Sausage [154]	Sausage [155] ^c^	Sausage [132]	Sausage [119]
	SPME (CAR/PDMS) ^b^	SPME (DVB/CAR/PDMS)	SPME (CAR/PDMS)	SPME (CAR/PDMS)	SPME (CAR/PDMS)	SPME (CAR/PDMS)	SAFE	SAFE
Methional	12,798.5	2.1	3.2	19.2		529.9		67.2
Methionol	20.6					5.5		
MT ^a^	0.4			0.14	147.9			
DMS	2.1			0.5				
DMDS	0.002			0.32	1730.8	0.5		
DMTS	3.9			0.07	104.1	2.5		
MFT	0.005			0.11		0.3	0.8	
MMTF				0		0.2		
MMFDS				0.27		0.1		
2AP	66.9					2.7	14	
APle	0.5					1.4		
2AT						0.2		
2A2T						0.7		
2-E-3,5-DMP							0.6	
2,3,5-TMP		2.4	2.8					
TMP		0.3	0.2					

^a^ Compound names are abbreviated as described in Figure 5 and Figure 6 except 2AT (2-acetylthiazole). ^b^ Volatile extraction method used for analysis of compounds: SPME (solid-phase micro-extraction), SAFE (solvent-assisted flavor evaporation). ^c^ Data are expressed in dry matter.

## Data Availability

No new data were created in this study. Data sharing is not applicable to this article.

## Supplementary Materials

The following supporting information can be downloaded at https://www.mdpi.com/article/10.3390/foods14050881/s1. Table S1: Aroma lexicon of dry-cured meat products [72,75,83,84,89,96,97]; Table S2: Savory and toasted aroma compounds detected in dry-cured meat products inoculated with yeast *D. hansenii* [70,82,129,145,147,156,157].

## References

[1] Flores M. (2018). Understanding the Implications of Current Health Trends on the Aroma of Wet and Dry Cured Meat Products. Meat Sci..

[2] (2021). Fermented Meat Product Specification.

[3] Vitale M., Kallas Z., Rivera-Toapanta E., Karolyi D., Cerjak M., Lebret B., Lenoir H., Pugliese C., Aquilani C., Čandek-Potokar M. (2020). Consumers’ Expectations and Liking of Traditional and Innovative Pork Products from European Autochthonous Pig Breeds. Meat Sci..

[4] Karolyi D., Škrlep M., Marušić Radovčić N., Luković Z., Škorput D., Salajpal K., Kljak K., Čandek-Potokar M. (2024). Effects of Animal Diet and Processing Methods on the Quality Traits of Dry-Cured Ham Produced from Turopolje Pigs. Animals.

[5] Belloch C., Neef A., Salafia C., López-Diez J.J., Flores M. (2021). Microbiota and Volatilome of Dry-Cured Pork Loins Manufactured with Paprika and Reduced Concentration of Nitrite and Nitrate. Food Res. Int..

[6] Vilar I., Garcia Fontan M.C., Prieto B., Tornadijo M.E., Carballo J. (2000). A Survey on the Microbiological Changes during the Manufacture of Dry-Cured Lacon, a Spanish Traditional Meat Product. J. Appl. Microbiol..

[7] Hao M., Wang W., Zhang J., Chen L. (2023). Flavour Characteristics of Fermented Meat Products in China: A Review. Fermentation.

[8] Li P., Bao Z., Wang Y., Su X., Zhou H., Xu B. (2023). Role of Microbiota and Its Ecological Succession on Flavor Formation in Traditional Dry-Cured Ham: A Review. Crit. Rev. Food Sci. Nutr..

[9] Jia D., Zhang J., Jin S., Luo S., Ma Y., Quek S.-Y., Yan D., Dong X. (2024). Changes of Physicochemical and Volatile Flavor Compounds of Dry-Cured Diqing Tibetan Pig Hams during Fermentation. Food Res. Int..

[10] Chen Y.P., Li W., Yu Y., Wang M., Blank I., Zhang Y., Liu Y. (2023). Elucidation of the Impact of Steaming on the Key Odorants of Jinhua Dry-Cured Ham Using the Sensomics Approach. J. Agric. Food Chem..

[11] Lebret B., Čandek-Potokar M. (2022). Review: Pork Quality Attributes from Farm to Fork. Part II. Processed Pork Products. Animal.

[12] Toledano A.M., Jordano R., Medina L.M., López-Mendoza M.C. (2019). Behavior and Effect of Combined Starter Cultures on Microbiological and Physicochemical Characteristics of Dry-Cured Ham. J. Food Sci. Technol..

[13] Pérez-Juan M., Flores M., Toldrá F. (2006). Generation of Volatile Flavour Compounds as Affected by the Chemical Composition of Different Dry-Cured Ham Sections. Eur. Food Res. Technol..

[14] Hernández P., Navarro J.L., Toldrá F. (1999). Lipolytic and Oxidative Changes in Two Spanish Pork Loin Products: Dry-Cured Loin and Pickled-Cured Loin. Meat Sci..

[15] Ramírez M.R., Cava R. (2007). Effect of Iberian × Duroc Genotype on Dry-Cured Loin Quality. Meat Sci..

[16] Soto E., Hoz L., Ordóñez J.A., Hierro E., Herranz B., López-Bote C., Cambero M.I. (2008). Impact of Feeding and Rearing Systems of Iberian Pigs on Volatile Profile and Sensory Characteristics of Dry-Cured Loin. Meat Sci..

[17] Martin D., Antequera T., Muriel E., Perez-Palacios T., Ruiz J. (2008). Effect of Dietary Conjugated Linoleic Acid in Combination with Monounsaturated Fatty Acids on the Meat Composition and Quality Traits of Dry-Cured Loin. Meat Sci..

[18] Armenteros M., Aristoy M.C., Barat J.M., Toldrá F. (2009). Biochemical Changes in Dry-Cured Loins Salted with Partial Replacements of NaCl by KCl. Food Chem..

[19] Soto E., De La Hoz L., Ordóñez J.A., Herranz B., Hierro E., López-Bote C.J., Cambero M.I. (2009). The Feeding and Rearing Systems of Iberian Pigs Affect the Lipid Composition and Texture Profile of Dry-Cured Loin. J. Anim. Feed Sci..

[20] Aliño M., Grau R., Toldrá F., Blesa E., Pagán M.J., Barat J.M. (2010). Physicochemical Properties and Microbiology of Dry-Cured Loins Obtained by Partial Sodium Replacement with Potassium, Calcium and Magnesium. Meat Sci..

[21] Salazar E., Cayuela J.M., Abellán A., Poto A., Peinado B., Tejada L. (2013). A Comparison of the Quality of Dry-Cured Loins Obtained from the Native Pig Breed (Chato Murciano) and from a Modern Crossbreed Pig. Anim. Prod. Sci..

[22] Pateiro M., Franco D., Carril J.A., Lorenzo J.M. (2015). Changes on Physico-Chemical Properties, Lipid Oxidation and Volatile Compounds during the Manufacture of Celta Dry-Cured Loin. J. Food Sci. Technol..

[23] Salazar E., Abellán A., Cayuela J.M., Poto Á., Tejada L. (2016). Dry-Cured Loin from the Native Pig Breed Chato Murciano with High Unsaturated Fatty Acid Content Undergoes Intense Lipolysis of Neutral and Polar Lipids during Processing. Eur. J. Lipid Sci. Technol..

[24] Cardoso-Toset F., Luque I., Morales-Partera A., Galán-Relaño A., Barrero-Domínguez B., Hernández M., Gómez-Laguna J. (2017). Survival of Streptococcus Suis, Streptococcus Dysgalactiae and Trueperella Pyogenes in Dry-Cured Iberian Pork Shoulders and Loins. Food Microbiol..

[25] Lorido L., Estévez M., Ventanas S. (2018). Fast and Dynamic Descriptive Techniques (Flash Profile, Time-Intensity and Temporal Dominance of Sensations) for Sensory Characterization of Dry-Cured Loins. Meat Sci..

[26] Higuero N., Moreno I., Lavado G., Vidal-Aragón M.C., Cava R. (2020). Reduction of Nitrate and Nitrite in Iberian Dry Cured Loins and Its Effects during Drying Process. Meat Sci..

[27] Seo J.-K., Ko J., Park J., Eom J.-U., Yang H.-S. (2021). Effect of Pig Breed and Processing Stage on the Physicochemical Properties of Dry-Cured Loin. Food Sci. Anim. Resour..

[28] Stadnik J., Dolatowski Z.J. (2013). Changes in Selected Parameters Related to Proteolysis during Ageing of Dry-Cured Pork Loins Inoculated with Probiotics. Food Chem..

[29] Stadnik J., Stasiak D.M. (2016). Effect of Acid Whey on Physicochemical Characteristics of Dry-Cured Organic Pork Loins without Nitrite. Int. J. Food Sci. Technol..

[30] Smagowska E., Derewiaka D., Jaworska D., Nowicka K., Przybylski W., Wołosiak R. (2019). Quality of Traditional Dry-Cured Loin from Rustic and Commercial Pig Breeds. Zesz. Probl. Postępów Nauk Rol..

[31] Zhang Y.W., Zhang L., Hui T., Guo X.Y., Peng Z.Q. (2015). Influence of Partial Replacement of NaCl by KCl, l-Histidine and l-Lysine on the Lipase Activity and Lipid Oxidation in Dry-Cured Loin Process. LWT Food Sci. Technol..

[32] Zhang J., Zhao K., Li H., Li S., Xu W., Chen L., Xie J., Tang H. (2023). Physicochemical Property, Volatile Flavor Quality, and Microbial Community Composition of Jinhua Fatty Ham and Lean Ham: A Comparative Study. Front. Microbiol..

[33] Seong P.N., Park K.M., Kang S.M., Kang G.H., Cho S.H., Park B.Y., Ba H.V. (2014). Effect of Particular Breed on the Chemical Composition, Texture, Color, and Sensorial Characteristics of Dry-Cured Ham. Asian-Australas. J. Anim. Sci..

[34] Kargozari M., Moini S., Akhondzadeh Basti A., Emam-Djomeh Z., Gandomi H., Revilla Martin I., Ghasemlou M., Carbonell-Barrachina Á.A. (2014). Effect of Autochthonous Starter Cultures Isolated from Siahmazgi Cheese on Physicochemical, Microbiological and Volatile Compound Profiles and Sensorial Attributes of Sucuk, a Turkish Dry-Fermented Sausage. Meat Sci..

[35] González-Fernández C., Santos E.M., Rovira J., Jaime I. (2006). The Effect of Sugar Concentration and Starter Culture on Instrumental and Sensory Textural Properties of Chorizo-Spanish Dry-Cured Sausage. Meat Sci..

[36] Van Reckem E., Geeraerts W., Charmpi C., Van der Veken D., De Vuyst L., Leroy F. (2019). Exploring the Link Between the Geographical Origin of European Fermented Foods and the Diversity of Their Bacterial Communities: The Case of Fermented Meats. Front. Microbiol..

[37] Encinas J.P., López-Díaz T.M., García-López M.L., Otero A., Moreno B. (2000). Yeast Populations on Spanish Fermented Sausages. Meat Sci..

[38] Leroy F., Charmpi C., De Vuyst L. (2023). Meat Fermentation at a Crossroads: Where the Age-Old Interplay of Human, Animal, and Microbial Diversity and Contemporary Markets Meet. Fems Microbiol. Rev..

[39] Roig-Sagués A.X., Hernández-Herrero M.M., López-Sabater E.I., Rodríguez-Jerez J.J., Mora-Ventura M.T. (1999). Microbiological Events during the Elaboration of “Fuet”, a Spanish Ripened sausageRelationships between the Development of Histidine- and Tyrosine-Decarboxylase-Containing Bacteria and pH and Water Activity. Eur. Food Res. Technol..

[40] Casquete R., Benito M.J., Martín A., Ruiz-Moyano S., Hernández A., Córdoba M.G. (2011). Effect of Autochthonous Starter Cultures in the Production of “Salchichón”, a Traditional Iberian Dry-Fermented Sausage, with Different Ripening Processes. LWT Food Sci. Technol..

[41] Ruiz-Moyano S., Martín A., Benito M.J., Hernández A., Casquete R., de Guia Córdoba M. (2011). Application of Lactobacillus Fermentum HL57 and Pediococcus Acidilactici SP979 as Potential Probiotics in the Manufacture of Traditional Iberian Dry-Fermented Sausages. Food Microbiol..

[42] Lorenzo J.M., González-Rodríguez R.M., Sánchez M., Amado I.R., Franco D. (2013). Effects of Natural (Grape Seed and Chestnut Extract) and Synthetic Antioxidants (Buthylatedhydroxytoluene, BHT) on the Physical, Chemical, Microbiological and Sensory Characteristics of Dry Cured Sausage “Chorizo”. Food Res. Int..

[43] Fonseca S., Cachaldora A., Gómez M., Franco I., Carballo J. (2013). Effect of Different Autochthonous Starter Cultures on the Volatile Compounds Profile and Sensory Properties of Galician Chorizo, a Traditional Spanish Dry Fermented Sausage. Food Control.

[44] Perea-Sanz L., Montero R., Belloch C., Flores M. (2019). Microbial Changes and Aroma Profile of Nitrate Reduced Dry Sausages during Vacuum Storage. Meat Sci..

[45] Dias I., Laranjo M., Potes M.E., Agulheiro-Santos A.C., Ricardo-Rodrigues S., Fialho A.R., Véstia J., Fraqueza M.J., Oliveira M., Elias M. (2020). Autochthonous Starter Cultures Are Able to Reduce Biogenic Amines in a Traditional Portuguese Smoked Fermented Sausage. Microorganisms.

[46] Belleggia L., Ferrocino I., Reale A., Corvaglia M.R., Milanović V., Cesaro C., Boscaino F., Di Renzo T., Garofalo C., Cardinali F. (2022). Unfolding Microbiota and Volatile Organic Compounds of Portuguese Painho de Porco Preto Fermented Sausages. Food Res. Int..

[47] Moretti V.M., Madonia G., Diaferia C., Mentasti T., Paleari M.A., Panseri S., Pirone G., Gandini G. (2004). Chemical and Microbiological Parameters and Sensory Attributes of a Typical Sicilian Salami Ripened in Different Conditions. Meat Sci..

[48] Patrignani F., Iucci L., Vallicelli M., Guerzoni M.E., Gardini F., Lanciotti R. (2007). Role of Surface-Inoculated Debaryomyces Hansenii and Yarrowia Lipolytica Strains in Dried Fermented Sausage Manufacture. Part 1: Evaluation of Their Effects on Microbial Evolution, Lipolytic and Proteolytic Patterns. Meat Sci..

[49] Casaburi A., Aristoy M.C., Cavella S., Di Monaco R., Ercolini D., Toldrá F., Villani F. (2007). Biochemical and Sensory Characteristics of Traditional Fermented Sausages of Vallo Di Diano (Southern Italy) as Affected by the Use of Starter Cultures. Meat Sci..

[50] Cenci-Goga B.T., Ranucci D., Miraglia D., Cioffi A. (2008). Use of Starter Cultures of Dairy Origin in the Production of Salame Nostrano, an Italian Dry-Cured Sausage. Meat Sci..

[51] Spaziani M., Torre M.D., Stecchini M.L. (2009). Changes of Physicochemical, Microbiological, and Textural Properties during Ripening of Italian Low-Acid Sausages. Proteolysis, Sensory and Volatile Profiles. Meat Sci..

[52] Tabanelli G., Coloretti F., Chiavari C., Grazia L., Lanciotti R., Gardini F. (2012). Effects of Starter Cultures and Fermentation Climate on the Properties of Two Types of Typical Italian Dry Fermented Sausages Produced under Industrial Conditions. Food Control.

[53] Tabanelli G., Montanari C., Grazia L., Lanciotti R., Gardini F. (2013). Effects of Aw at Packaging Time and Atmosphere Composition on Aroma Profile, Biogenic Amine Content and Microbiological Features of Dry Fermented Sausages. Meat Sci..

[54] Coloretti F., Tabanelli G., Chiavari C., Lanciotti R., Grazia L., Gardini F., Montanari C. (2014). Effect of Wine Addition on Microbiological Characteristics, Volatile Molecule Profiles and Biogenic Amine Contents in Fermented Sausages. Meat Sci..

[55] Christieans S., Picgirard L., Parafita E., Lebert A., Gregori T. (2018). Impact of Reducing Nitrate/Nitrite Levels on the Behavior of Salmonella Typhimurium and Listeria Monocytogenes in French Dry Fermented Sausages. Meat Sci..

[56] Janssens M., Myter N., De Vuyst L., Leroy F. (2012). Species Diversity and Metabolic Impact of the Microbiota Are Low in Spontaneously Acidified Belgian Sausages with an Added Starter Culture of Staphylococcus Carnosus. Food Microbiol..

[57] Hagen B.F., Berdagué J.L., Holck A.L., Næs H., Blom H. (1996). Bacterial Proteinase Reduces Maturation Time of Dry Fermented Sausages. J. Food Sci..

[58] Xiao Y., Liu Y., Chen C., Xie T., Li P. (2020). Effect of Lactobacillus Plantarum and Staphylococcus Xylosus on Flavour Development and Bacterial Communities in Chinese Dry Fermented Sausages. Food Res. Int..

[59] Hu Y., Wang H., Kong B., Wang Y., Chen Q. (2021). The Succession and Correlation of the Bacterial Community and Flavour Characteristics of Harbin Dry Sausages during Fermentation. LWT.

[60] Chen X., Mi R., Qi B., Xiong S., Li J., Qu C., Qiao X., Chen W., Wang S. (2021). Effect of Proteolytic Starter Culture Isolated from Chinese Dong Fermented Pork (Nanx Wudl) on Microbiological, Biochemical and Organoleptic Attributes in Dry Fermented Sausages. Food Sci. Hum. Wellness.

[61] Liu Y., Wan Z., Yohannes K.W., Yu Q., Yang Z., Li H., Liu J., Wang J. (2021). Functional Characteristics of Lactobacillus and Yeast Single Starter Cultures in the Ripening Process of Dry Fermented Sausage. Front. Microbiol..

[62] Wang J., Hou J., Zhang X., Hu J., Yu Z., Zhu Y. (2022). Improving the Flavor of Fermented Sausage by Increasing Its Bacterial Quality via Inoculation with Lactobacillus Plantarum MSZ2 and Staphylococcus Xylosus YCC3. Foods.

[63] Bleicher J., Ebner E.E., Bak K.H. (2022). Formation and Analysis of Volatile and Odor Compounds in Meat—A Review. Molecules.

[64] Li L., Belloch C., Flores M. Generation of Meaty Flavour Compounds from Maillard Origin under Mild Conditions Simulating the Dry Curing Process. Proceedings of the 16th Weurman Flavour Research Symposium.

[65] Flores M., Corral S., Toldrá F., Nollet L.M.L. (2017). Olfactometry Detection of Aroma Compounds. Advances in Food Diagnostics.

[66] Song H., Cadwallader K.R., Singh T.K. (2008). Odour-Active Compounds of Jinhua Ham. Flavour Fragr. J..

[67] Škrlep M., Čandek-Potokar M., Lukač N.B., Povše M.P., Pugliese C., Labussière E., Flores M. (2016). Comparison of Entire Male and Immunocastrated Pigs for Dry-Cured Ham Production under Two Salting Regimes. Meat Sci..

[68] Muriel E., Antequera T., Petrón M.J., Andrés A.I., Ruiz J. (2004). Volatile Compounds in Iberian Dry-Cured Loin. Meat Sci..

[69] Muriel E., Antequera T., Petrón M.J., Martin D., Ruiz J. (2004). Volatile Compounds on the Surface and within Iberian Dry-Cured Loin. Eur. Food Res. Technol..

[70] Corral S., Salvador A., Belloch C., Flores M. (2015). Improvement the Aroma of Reduced Fat and Salt Fermented Sausages by Debaromyces Hansenii Inoculation. Food Control.

[71] Corral S., Salvador A., Flores M. (2016). Effect of the Use of Entire Male Fat in the Production of Reduced Salt Fermented Sausages. Meat Sci..

[72] González-Mohino A., Ventanas S., Estévez M., Olegario L.S. (2021). Sensory Characterization of Iberian Dry-Cured Loins by Using Check-All-That-Apply (CATA) Analysis and Multiple-Intake Temporal Dominance of Sensations (TDS). Foods.

[73] Górska E., Nowicka K., Jaworska D., Przybylski W., Tambor K. (2017). Relationship between Sensory Attributes and Volatile Compounds of Polish Dry-Cured Loin. Asian-Australas. J. Anim. Sci..

[74] Berdagué J.L., Monteil P., Montel M.C., Talon R. (1993). Effects of Starter Cultures on the Formation of Flavour Compounds in Dry Sausage. Meat Sci..

[75] Armero E., Flores M., Toldrá F., Barbosa J.-A., Olivet J., Pla M., Baselga M. (1999). Effects of Pig Sire Type and Sex on Carcass Traits, Meat Quality and Sensory Quality of Dry-Cured Ham. J. Sci. Food Agric..

[76] Buscailhon S., Touraille C., Girard J.P., Monin G. (1995). Relationships between Muscle Tissue Characteristics and Sensory Qualities of Dry-Cured Ham. J. Muscle Foods.

[77] Buscailhon S., Berdagué J.L., Bousset J., Cornet M., Gandemer G., Touraille C., Monin G. (1994). Relations between Compositional Traits and Sensory Qualities of French Dry-Cured Ham. Meat Sci..

[78] Carrapiso A.I., Jurado Á., Timón M.L., García C. (2002). Odor-Active Compounds of Iberian Hams with Different Aroma Characteristics. J. Agric. Food Chem..

[79] Cilla I., Martínez L., Beltrán J.A., Roncalés P. (2005). Factors Affecting Acceptability of Dry-Cured Ham throughout Extended Maturation under “Bodega” Conditions. Meat Sci..

[80] Corino C., Magni S., Pastorelli G., Rossi R., Mourot J. (2003). Effect of Conjugated Linoleic Acid on Meat Quality, Lipid Metabolism, and Sensory Characteristics of Dry-Cured Hams from Heavy Pigs1. J. Anim. Sci..

[81] García-González D.L., Tena N., Aparicio-Ruiz R., Morales M.T. (2008). Relationship between Sensory Attributes and Volatile Compounds Qualifying Dry-Cured Hams. Meat Sci..

[82] Martín A., Córdoba J.J., Aranda E., Córdoba M.G., Asensio M.A. (2006). Contribution of a Selected Fungal Population to the Volatile Compounds on Dry-Cured Ham. Int. J. Food Microbiol..

[83] Pagliarini E., Laureati M., Dinnella C., Monteleone E., Proserpio C., Piasentier E. (2016). Influence of Pig Genetic Type on Sensory Properties and Consumer Acceptance of Parma, San Daniele and Toscano Dry-Cured Hams. J. Sci. Food Agric..

[84] Pham A.J., Schilling M.W., Mikel W.B., Williams J.B., Martin J.M., Coggins P.C. (2008). Relationships between Sensory Descriptors, Consumer Acceptability and Volatile Flavor Compounds of American Dry-Cured Ham. Meat Sci..

[85] Ruiz J., Ventanas J., Cava R., Timón M.L., García C. (1998). Sensory Characteristics of Iberian Ham: Influence of Processing Time and Slice Location. Food Res. Int..

[86] Sánchez-Molinero F., Arnau J. (2008). Effect of the Inoculation of a Starter Culture and Vacuum Packaging during the Resting Stage on Sensory Traits of Dry-Cured Ham. Meat Sci..

[87] Schivazappa C., Virgili R. (2020). Impact of Salt Levels on the Sensory Profile and Consumer Acceptance of Italian Dry-Cured Ham. J. Sci. Food Agric..

[88] Segura-Borrego M.P., Ríos-Reina R., Galán-Soldevilla H., Forero F.J., Venegas M., Ruiz Pérez-Cacho P., Morales M.L., Callejón R.M. (2022). Influence of the Ripening Chamber’s Geographical Location on Dry-Cured Iberian Ham’s Key Odorants. Food Res. Int..

[89] Simoncini N., Pinna A., Toscani T., Virgili R. (2015). Effect of Added Autochthonous Yeasts on the Volatile Compounds of Dry-Cured Hams. Int. J. Food Microbiol..

[90] Gamero-Negrón R., García C., Reina R., Sánchez del Pulgar J. (2018). Immune-Spaying as an Alternative to Surgical Spaying in Iberian x Duroc Females: Effect on the Sensory Traits and Volatile Organic Compound Profile of Dry-Cured Shoulders and Dry-Cured Loins. Meat Sci..

[91] Jiménez A., González-Mohino A., Rufo M., Paniagua J., Antequera T., Pérez-Palacios T. (2022). Dry-Cured Loin Characterization by Ultrasound Physicochemical and Sensory Parameters. Eur. Food Res. Technol..

[92] Lorido L., Ventanas S., Akcan T., Estévez M. (2016). Effect of Protein Oxidation on the Impaired Quality of Dry-Cured Loins Produced from Frozen Pork Meat. Food Chem..

[93] Lušnic Polak M., Polak T., Dolhar U., Demšar L. (2018). Effect of Iodized Salt on the Physicochemical Parameters and the Sensory Properties of Dry-Cured Pork Loin. MESO Prvi Hrvat. Časopis O Mesu.

[94] Muriel E., Ruiz J., Martin D., Petron M.J., Antequera T. (2004). Physico-Chemical and Sensory Characteristics of Dry-Cured Loin from Different Iberian Pig Lines. Food Sci. Technol. Int..

[95] Palavecino Prpich N.Z., Castro M.P., Cayré M.E., Garro O.A., Vignolo G.M. (2015). Indigenous Starter Cultures to Improve Quality of Artisanal Dry Fermented Sausages from Chaco (Argentina). Int. J. Food Sci..

[96] Pérez-Cacho M.P.R., Galán-Soldevilla H., Crespo F.L., Recio G.M. (2005). Determination of the Sensory Attributes of a Spanish Dry-Cured Sausage. Meat Sci..

[97] Rason J., Laguet A., Berge P., Dufour E., Lebecque A. (2007). Investigation of the Physicochemical and Sensory Homogeneity of Traditional French Dry Sausages. Meat Sci..

[98] Stahnke L.H. (1995). Dried Sausages Fermented with Staphylococcus Xylosus at Different Temperatures and with Different Ingredient Levels—Part III. Sensory Evaluation. Meat Sci..

[99] Stahnke L.H., Holck A., Jensen A., Nilsen A., Zanardi E. (2002). Maturity Acceleration of Italian Dried Sausage by Staphylococcus Carnosus—Relationship Between Maturity and Flavor Compounds. J. Food Sci..

[100] Zinina O., Merenkova S., Soloveva A., Savostina T., Sayfulmulyukov E., Lykasova I., Mizhevikina A. (2018). The Effect of Starter Cultures on the Qualitative Indicators of Dry Fermented Sausages Made from Poultry Meat. Agron. Res..

[101] Baldovini N., Chaintreau A. (2020). Identification of Key Odorants in Complex Mixtures Occurring in Nature. Nat. Prod. Rep..

[102] Marsili R., Marsili R. (2006). Application of Sensory- Directed Flavor- Analysis Techniques. Sensory-Directed Flavor Analysis.

[103] Narváez-Rivas M., Gallardo E., León-Camacho M. (2012). Analysis of Volatile Compounds from Iberian Hams: A Review. Grasas Y Aceites.

[104] Li L., Perea-Sanz L., Salvador A., Belloch C., Flores M. (2022). Understanding the Impact of Nitrogen and Sulfur Precursors on the Aroma of Dry Fermented Sausages. Meat Sci..

[105] Yan Y., Chen S., Nie Y., Xu Y. (2021). Quantitative Analysis of Pyrazines and Their Perceptual Interactions in Soy Sauce Aroma Type Baijiu. Foods.

[106] Chen Y.P., Wang M., Fang X., Liya A., Zhang H., Blank I., Zhu H., Liu Y. (2024). Odorants Identified in Chinese Dry-Cured Ham Contribute to Salty Taste Enhancement. J. Agric. Food Chem..

[107] Carrapiso A.I., Ventanas J., García C. (2002). Characterization of the Most Odor-Active Compounds of Iberian Ham Headspace. J. Agric. Food Chem..

[108] Carrapiso A.I., García C. (2004). Iberian Ham Headspace: Odorants of Intermuscular Fat and Differences with Lean. J. Sci. Food Agric..

[109] Carrapiso A.I., Martín L., Jurado Á., García C. (2010). Characterisation of the Most Odour-Active Compounds of Bone Tainted Dry-Cured Iberian Ham. Meat Sci..

[110] Del Pulgar J.S., García C., Reina R., Carrapiso A.I. (2013). Study of the Volatile Compounds and Odor-Active Compounds of Dry-Cured Iberian Ham Extracted by SPME. Food Sci. Technol. Int..

[111] Flores M., Grimm C.C., Toldrá F., Spanier A.M. (1997). Correlations of Sensory and Volatile Compounds of Spanish “Serrano” Dry-Cured Ham as a Function of Two Processing Times. J. Agric. Food Chem..

[112] Liu X.S., Liu J.B., Yang Z.M., Song H.L., Liu Y., Zou T.T. (2014). Aroma-Active Compounds in Jinhua Ham Produced with Different Fermentation Periods. Molecules.

[113] Li L., Perea-Sanz L., López-Díez J.J., Salvador A., Belloch C., Flores M. (2022). Aroma Enhancement in Dry Cured Loins by the Addition of Nitrogen and Sulfur Precursors. Meat Sci..

[114] Sekhon R.K., Schilling M.W., Phillips T.W., Aikins R.M.J., Hasan M.M., Nannapaneni R., Mikel W.B. (2010). Effects of Carbon Dioxide and Ozone Treatments on the Volatile Composition and Sensory Quality of Dry-cured Ham. J. Food Sci..

[115] Song H., Cadwallader K.R. (2008). Aroma Components of American Country Ham. J. Food Sci..

[116] Théron L., Tournayre P., Kondjoyan N., Abouelkaram S., Santé-Lhoutellier V., Berdagué J.L. (2010). Analysis of the Volatile Profile and Identification of Odour-Active Compounds in Bayonne Ham. Meat Sci..

[117] Aquilani C., Sirtori F., Flores M., Bozzi R., Lebret B., Pugliese C. (2018). Effect of Natural Antioxidants from Grape Seed and Chestnut in Combination with Hydroxytyrosol, as Sodium Nitrite Substitutes in Cinta Senese Dry-Fermented Sausages. Meat Sci..

[118] Corral S., Salvador A., Flores M. (2013). Salt Reduction in Slow Fermented Sausages Affects the Generation of Aroma Active Compounds. Meat Sci..

[119] Corral S., Salvador A., Flores M. (2014). Elucidation of Key Aroma Compounds in Traditional Dry Fermented Sausages Using Different Extraction Techniques. J. Sci. Food Agric..

[120] Corral S., Leitner E., Siegmund B., Flores M. (2016). Determination of Sulfur and Nitrogen Compounds during the Processing of Dry Fermented Sausages and Their Relation to Amino Acid Generation. Food Chem..

[121] Corral S., Belloch C., López-Díez J.J., Flores M. (2018). Lipolysis and Aroma Generation as Mechanisms Involved in Masking Boar Taint in Sodium Reduced Fermented Sausages Inoculated with Debaryomyces Hansenii Yeast. J. Sci. Food Agric..

[122] Gianelli M.P., Olivares A., Flores M. (2011). Key Aroma Components of a Dry-Cured Sausage with High Fat Content (Sobrassada). Food Sci. Technol. Int..

[123] Marco A., Navarro J.L., Flores M. (2007). Quantitation of Selected Odor-Active Constituents in Dry Fermented Sausages Prepared with Different Curing Salts. J. Agric. Food Chem..

[124] Meynier A., Novelli E., Chizzolini R., Zanardi E., Gandemer G. (1999). Volatile Compounds of Commercial Milano Salami. Meat Sci..

[125] Olivares A., Dryahina K., Navarro J.L., Flores M., Smith D., Španěl P. (2010). Selected Ion Flow Tube-Mass Spectrometry for Absolute Quantification of Aroma Compounds in the Headspace of Dry Fermented Sausages. Anal. Chem..

[126] Olivares A., Navarro J.L., Flores M. (2011). Effect of Fat Content on Aroma Generation during Processing of Dry Fermented Sausages. Meat Sci..

[127] Olivares A., Navarro J.L., Flores M. (2015). Characterization of Volatile Compounds Responsible for the Aroma in Naturally Fermented Sausages by Gas Chromatography-Olfactometry. Food Sci. Technol. Int..

[128] Perea-Sanz L., Montero R., Belloch C., Flores M. (2018). Nitrate Reduction in the Fermentation Process of Salt Reduced Dry Sausages: Impact on Microbial and Physicochemical Parameters and Aroma Profile. Int. J. Food Microbiol..

[129] Perea-Sanz L., López-Díez J.J., Belloch C., Flores M. (2020). Counteracting the Effect of Reducing Nitrate/Nitrite Levels on Dry Fermented Sausage Aroma by Debaryomyces Hansenii Inoculation. Meat Sci..

[130] Stahnke L.H. Character Impact Aroma Compounds in Fermented Sausage. Proceedings of the 44th ICoMST.

[131] Schmidt S., Berger R.G. (1998). Aroma Compounds in Fermented Sausages of Different Origins. LWT-Food Sci. Technol..

[132] Söllner K., Schieberle P. (2009). Decoding the Key Aroma Compounds of a Hungarian-Type Salami by Molecular Sensory Science Approaches. J. Agric. Food Chem..

[133] Rowe D. (1998). Aroma Chemicals for Savory Flavors. Perfum. Flavorist.

[134] Mottram D.S. (1998). Flavour Formation in Meat and Meat Products: A Review. Food Chem..

[135] Parker J.K., Buettner A. (2017). Meat. Springer Handbook of Odor.

[136] Li L., Belloch C., Flores M. (2023). Short-Term Changes in Aroma-Related Volatiles in Meat Model: Effect of Fat and D. Hansenii Inoculation. Foods.

[137] Resconi V.C., Escudero A., Campo M.M. (2013). The Development of Aromas in Ruminant Meat. Molecules.

[138] Hofmann T., Schieberle P. (1998). 2-Oxopropanal, Hydroxy-2-Propanone, and 1-PyrrolineImportant Intermediates in the Generation of the Roast-Smelling Food Flavor Compounds 2-Acetyl-1-Pyrroline and 2-Acetyltetrahydropyridine. J. Agric. Food Chem..

[139] Daygon V.D., Calingacion M., Forster L.C., De Voss J.J., Schwartz B.D., Ovenden B., Alonso D.E., McCouch S.R., Garson M.J., Fitzgerald M.A. (2017). Metabolomics and Genomics Combine to Unravel the Pathway for the Presence of Fragrance in Rice. Sci. Rep..

[140] Li L., Belloch C., Flores M. (2021). The Maillard Reaction as Source of Meat Flavor Compounds in Dry Cured Meat Model Systems under Mild Temperature Conditions. Molecules.

[141] Li L., Belloch C., Flores M. (2023). A Comparative Study of Savory and Toasted Aromas in Dry Cured Loins versus Dry Fermented Sausages. LWT.

[142] Schieberle P. (1990). The Role of Free Amino Acids Present in Yeast as Precursors of the Odorants 2-Acetyl-1-Pyrroline and 2-Acetyltetrahydropyridine in Wheat Bread Crust. Z Leb. Unters Forch.

[143] Carballo J., Mehta B.M., Kamal-Eldin A., Iwanski R.Z. (2012). The Role of Fermentation Reactions in the Generation of Flavor and Aroma of Foods. Fermentation, Effects on Food Properties.

[144] Beck H.C., Hansen A.M., Lauritsen F.R. (2002). Metabolite Production and Kinetics of Branched-Chain Aldehyde Oxidation in Staphylococcus Xylosus. Enzym. Microb. Technol..

[145] Andrade M.J., Rodríguez M., Casado E.M., Bermúdez E., Córdoba J.J. (2009). Differentiation of Yeasts Growing on Dry-Cured Iberian Ham by Mitochondrial DNA Restriction Analysis, RAPD-PCR and Their Volatile Compounds Production. Food Microbiol..

[146] Andrade M.J., Córdoba J.J., Casado E.M., Córdoba M.G., Rodríguez M. (2010). Effect of Selected Strains of Debaryomyces Hansenii on the Volatile Compound Production of Dry Fermented Sausage “salchichón”. Meat Sci..

[147] Martín A., Córdoba J.J., Benito M.J., Aranda E., Asensio M.A. (2003). Effect of Penicillium Chrysogenum and Debaryomyces Hansenii on the Volatile Compounds during Controlled Ripening of Pork Loins. Int. J. Food Microbiol..

[148] Perea-Sanz L., Peris D., Belloch C., Flores M. (2019). Debaryomyces Hansenii Metabolism of Sulfur Amino Acids As Precursors of Volatile Sulfur Compounds of Interest in Meat Products. J. Agric. Food Chem..

[149] Belloch C., Perea-Sanz L., Gamero A., Flores M. (2022). Selection of Debaryomyces Hansenii Isolates as Starters in Meat Products Based on Phenotypic Virulence Factors, Tolerance to Abiotic Stress Conditions and Aroma Generation. J. Appl. Microbiol..

[150] Stahnke L., Schieberle P., Engel K.H. (2000). 2-Acetyl-1-Pyrroline–Key Aroma Compound in Mediterranean Dried Sausages. Frontiers in Flavour Science.

[151] Flores M., Olivares A., Toldrá F., Hui Y.H., Astiasarán I., G. Sebranek J., Talon R. (2014). Flavor. Handbook of Fermented Meat and Poultry.

[152] Jeleń H., Majcher M., Gracka A., Ouyang G., Jiang R. (2017). Application of Solid Phase Microextraction in Food Analysis—Flavor and Off-Flavor Sampling. Solid Phase Microextraction: Recent Developments and Applications.

[153] Varlet V., Fernandez X. (2010). Review. Sulfur-Containing Volatile Compounds in Seafood: Occurrence, Odorant Properties and Mechanisms of Formation. Food Sci. Technol. Int..

[154] Cano-García L., Rivera-Jiménez S., Belloch C., Flores M. (2014). Generation of Aroma Compounds in a Fermented Sausage Meat Model System by Debaryomyces Hansenii Strains. Food Chem..

[155] Flores M., Perea-Sanz L., López-Díez J.J., Belloch C. (2021). Meaty Aroma Notes from Free Amino Acids and Thiamine in Nitrite-Reduced, Dry-Fermented, Yeast-Inoculated Sausages. Food Chem..

[156] Ramos-Moreno L., Ruiz-Castilla F.J., Bravo C., Martínez E., Menéndez M., Dios-Palomares R., Ramos J. (2019). Inoculation with a Terroir Selected Debaryomyces Hansenii Strain Changes Physico-Chemical Characteristics of Iberian Cured Pork Loin. Meat Sci..

[157] Cano-García L., Belloch C., Flores M. (2014). Impact of Debaryomyces Hansenii Strains Inoculation on the Quality of Slow Dry-Cured Fermented Sausages. Meat Sci..

