# Supplementary material for: The Aroma of Non-Fermented and Fermented Dry-Cured Meat Products: Savory and Toasted Odors
Source: Foods. 2025 Mar 5;14(5):881. doi: 10.3390/foods14050881 (PMC11899295; doi:10.3390/foods14050881)
Supplement: Supplementary file 1 [file foods-14-00881-s001.zip › foods-3344511-supplementary.pdf]

Review

The Aroma of Non-Fermented and Fermented Dry-Cured Meat Products: Savory and Toasted Odors

Lei Li, Carmela Belloch and Mónica Flores *

Institute of Agrochemistry and Food Technology (IATA-CSIC), Agustín Escardino Avenue 7, 46980 Paterna, Valencia, Spain
*   Correspondence: mflores@iata.csic.es

Table S1. Aroma lexicon of dry-cured meat products.

Products	Modality/attribute	Definition	Ref*
Ham	Cured ham [1,2]	Aroma of long-matured dried ham	1,2
	Dry [1]	Dust- and mite-type smell associated with excessively dried ham	1
	Rancid [1–3]	Odor associated with lipid autooxidation	1,2,3
	Cheesy [1]	Odor of cheese resulting from abnormal yeast growth	1
	Musty [1]	Mold-type smell resulting from abnormal fungi growth	1
	Molasses [3]	Aroma associated with molasses; has sharp, slight sulfur and/or caramelized character	3
	Fermented [3]	Aroma associated with lactic or spoilage bacteria as in soured meat	3
	Caramelized [3]	Sweet aroma, characteristic of browned sugars and some other carbohydrates	3
	Pork complex [3]	Aroma associated with live pig or its habitat or wet pig hair	3
	Smoky [3]	Perception of any type of smoke aroma	3
	Earthy [3]	Aroma characteristic of damp soil, wet foliage, or slightly undercooked boiled potato	3
	Savory [3]	Aroma associated with savoriness or meatiness	3
	Fat complex [4]	Aroma associated with lipid products such as animal fat and lard	4
	Barnyard [4]	Aroma associated with free fatty acids	4
	Pickling spice [4]	Aroma associated with dill and vinegar	4
	Nutty [4]	Aroma associated with nuts and almonds	4
	Rancid [4]	Aroma associated with extremely oxidized fat or oil	4
	Serum [4]	Aroma associated with raw lean meat and cooked blood	4
	Pork [2,4]	Aroma associated with cooked pork muscle meat	2, 4
Loin	Rancid [5,6]	Odor associated with oxidation compounds derived from fat or old butter	5, 6
	Paprika [7]	Odor perception characteristic of paprika	7
	Marinade [6]	Aroma associated with meat marinated in wine	6

	Seasoning, spicy, and herbal [7]	Odor perception characteristic of pepper, garlic, and nutmeg	7
	Fatty [5]	Odor associated with volatile compounds released by exudate fat	5
	Sour [5]	Describes olfactory complex sensation, generally due to presence of organic acids	5
Sausage	Black pepper [5]	Odor associated with black pepper	5
	Lactic acid [5]	Characteristics odor of lactic acid, related to yogurt	5
	Mold [5]	Characteristic odor associated with chemical compound 1-octen-3-ol, related to mushroom odor	5
	Spices [5]	Odor associated with aromatic spices added to salchichon (nutmeg, anise, cumin, cinnamon, etc.) which are different from black pepper	5
	Vinegar [6]	Aroma associated with vinegar	6
	Salting [6]	Aroma associated with salting department	6
	Roast [6]	Aroma associated with roasted pork	6
	Salami [5]	Sour characteristic odor that presents salami which is related to lactic acid	5

Table S2. Savory and toasted aroma compounds detected in dry-cured meat products inoculated with yeast *D. hansenii*.

	**Compounds**	**Dry-cured ham**		**Dry-cured loins**		**Dry-fermented sausages**		
		**Dry-cured ham** [8]	**Dry-cured Iberian ham** [9]	**Dry-cured pork loins** [10]	**Dry-cured Iberian pork loins** [11]	**Slow dry-fermented sausages** [12]	**Dry-fermented sausages [13]**	**Dry-fermented sausages [14]**
Savory aroma	Methional	+	+		+		+	+
	Methionol		+			+	+	
	Methanethiol	+	+	+	+	+	+	+
	Dimethyl sulfide	+		+			+	
	Dimethyl disulfide	+	+	+		+	+	+
	Dimethyl trisulfide		+			+		+
	2-Methyl-3-furanthiol							+
	Methyl 2-methyl-3-furyl disulfide							+
Toasted aroma	2-Acetyl-1-pyrroline						+	+
	2-Acetyl-2-thiazoline							+
	Methylpyrazine	+		+			+	
	2,5-Dimethylpyrazine	+		+				
	2,6-Dimethylpyrazine						+	+
	2-Ethyl-3,5-dimethylpyrazine			+				
	2,3,5-Trimethyl-pyrazine	+		+				
	Tetramethylpyrazine			+				

Reference:

1. Simoncini, N.; Pinna, A.; Toscani, T.; Virgili, R. Effect of Added Autochthonous Yeasts on the Volatile Compounds of Dry-Cured Hams. *Int. J. Food Microbiol.* **2015**, *212*, 25–33, doi:10.1016/j.ijfoodmicro.2015.06.024.
2. Pagliarini, E.; Laureati, M.; Dinnella, C.; Monteleone, E.; Proserpio, C.; Piasentier, E. Influence of Pig Genetic Type on Sensory Properties and Consumer Acceptance of Parma, San Daniele and Toscano Dry-Cured Hams. *J. Sci. Food Agric.* **2016**, *96*, 798–806, doi:10.1002/jsfa.7151.
3. Pham, A.J.; Schilling, M.W.; Mikel, W.B.; Williams, J.B.; Martin, J.M.; Coggins, P.C. Relationships between Sensory Descriptors, Consumer Acceptability and Volatile Flavor Compounds of American Dry-Cured Ham. *Meat Sci.* **2008**, *80*, 728–737, doi:10.1016/j.meatsci.2008.03.015.
4. Armero, E.; Flores, M.; Toldrá, F.; Barbosa, J.-A.; Olivet, J.; Pla, M.; Baselga, M. Effects of Pig Sire Type and Sex on Carcass Traits, Meat Quality and Sensory Quality of Dry-Cured Ham. *J. Sci. Food Agric.* **1999**, *79*, 1147–1154, doi:10.1002/(SICI)1097-0010(19990701)79:9<1147::AID-JSFA340>3.0.CO;2-F.
5. Pérez-Cacho, M.P.R.; Galán-Soldevilla, H.; Crespo, F.L.; Recio, G.M. Determination of the Sensory Attributes of a Spanish Dry-Cured Sausage. *Meat Sci.* **2005**, *71*, 620–633, doi:10.1016/j.meatsci.2005.05.005.
6. Rason, J.; Laguet, A.; Berge, P.; Dufour, E.; Lebecque, A. Investigation of the Physicochemical and Sensory Homogeneity of Traditional French Dry Sausages. *Meat Sci.* **2007**, *75*, 359–370, doi:10.1016/j.meatsci.2006.06.004.
7. González-Mohino, A.; Ventanas, S.; Estévez, M.; Olegario, L.S. Sensory Characterization of Iberian Dry-Cured Loins by Using Check-All-That-Apply (CATA) Analysis and Multiple-Intake Temporal Dominance of Sensations (TDS). *Foods* **2021**, *10*, 1983, doi:10.3390/foods10091983.
8. Martín, A.; Córdoba, J.J.; Aranda, E.; Córdoba, M.G.; Asensio, M.A. Contribution of a Selected Fungal Population to the Volatile Compounds on Dry-Cured Ham. *Int. J. Food Microbiol.* **2006**, *110*, 8–18, doi:10.1016/j.ijfoodmicro.2006.01.031.
9. Andrade, M.J.; Rodríguez, M.; Casado, E.M.; Bermúdez, E.; Córdoba, J.J. Differentiation of Yeasts Growing on Dry-Cured Iberian Ham by Mitochondrial DNA Restriction Analysis, RAPD-PCR and Their Volatile Compounds Production. *Food Microbiol.* **2009**, *26*, 578–586, doi:10.1016/j.fm.2009.03.014.
10. Martín, A.; Córdoba, J.J.; Benito, M.J.; Aranda, E.; Asensio, M.A. Effect of Penicillium Chrysogenum and Debaryomyces Hansenii on the Volatile Compounds during Controlled Ripening of Pork Loins. *Int. J. Food Microbiol.* **2003**, *84*, 327–338, doi:10.1016/S0168-1605(02)00474-9.
11. Ramos-Moreno, L.; Ruiz-Castilla, F.J.; Bravo, C.; Martínez, E.; Menéndez, M.; Dios-Palomares, R.; Ramos, J. Inoculation with a Terroir Selected Debaryomyces Hansenii Strain Changes Physico-Chemical Characteristics of Iberian Cured Pork Loin. *Meat Sci.* **2019**, *157*, 107875, doi:10.1016/j.meatsci.2019.107875.
12. Cano-García, L.; Belloch, C.; Flores, M. Impact of Debaryomyces Hansenii Strains Inoculation on the Quality of Slow Dry-Cured Fermented Sausages. *Meat Sci.* **2014**, *96*, 1469–1477, doi:10.1016/j.meatsci.2013.12.011.
13. Corral, S.; Salvador, A.; Belloch, C.; Flores, M. Improvement the Aroma of Reduced Fat and Salt Fermented Sausages by Debaromyces Hansenii Inoculation. *Food Control* **2015**, *47*, 526–535,

doi:10.1016/j.foodcont.2014.08.001.
14. Perea-Sanz, L.; López-Díez, J.J.; Belloch, C.; Flores, M. Counteracting the Effect of Reducing Nitrate/Nitrite Levels on Dry Fermented Sausage Aroma by Debaryomyces Hansenii Inoculation. *Meat Sci.* **2020**, *164*, 108103, doi:10.1016/j.meatsci.2020.108103.
